# Deficiencies in Root Canal Fillings Subsequent to Adaptive Instrumentation of Oval Canals

**DOI:** 10.3390/biology10111074

**Published:** 2021-10-21

**Authors:** Ajinkya M. Pawar, Anuj Bhardwaj, Kulvinder S. Banga, Gurdeep Singh, Anda Kfir, Alexander Maniangat Luke, Vialyne Dinata, Dian Agustin Wahjuningrun

**Affiliations:** 1 Department of Conservative Dentistry and Endodontics, Nair Hospital Dental College, Mumbai 400008, India; ajinkya@drpawars.com (A.M.P.); ksbanga@gmail.com (K.S.B.); 2 Department of Conservative Dentistry and Endodontics, College of Dental Sciences & Hospital, Rau, Indore 453331, India; dranuj_84@yahoo.co.in; 3 Department of Adult Restorative Dentistry, Oman Dental College, P.O. Box 835, Mina Al Fahal, Muscat 116, Oman; gsingh@staff.omandentalcollege.org; 4 Department of Endodontology, The Goldschleger School of Dental Medicine, Tel Aviv University, Tel Aviv 69978, Israel; dr.andakfir@gmail.com; 5 Department of Clinical Science, College of Dentistry, Ajman University, Al-Jurf, Ajman 346, United Arab Emirates; 6 Center of Medical and Bio-Allied Health Sciences Research, Ajman University, Al-Jurf, Ajman 346, United Arab Emirates; 7 Department of Conservative Dentistry, Faculty of Dental Medicine, Universitas Airlingga, Surabaya 60132, Indonesia; vialyne_11@yahoo.com

**Keywords:** adaptive files, debris, root canal instrumentation, self-adjusting files, XP-endo files

## Abstract

**Simple Summary:**

Employing a chemo-mechanical preparation, one of the primary procedural phases in endodontic therapy is carefully removing debris, pulp tissue, and bacteria from the root canal system. The cross-sectional root canal shapes comprise circular, oval, long oval, flattened, or irregular. The frequency of oval root canals in the apical third of human teeth is around 25 to 50%. Motorised endodontic files leave almost 60% of the oval root canal perimeter unaffected by their instrumentation, thus resulting in faulty obturation that is required to prevent reinfection and to restrict the passage of microorganisms and toxins to and from the periapical tissue.

**Abstract:**

The purpose of this study was to explore the influence of instrumentation and the potential for debris deposition using XP-endo shaper plus (XP-SP) and full-sequence SAF (F-SAF) on the adaption of thermoplastic root canal fillings in oval canals. Following the manufacturer’s instructions, ninety human permanent mandibular incisors with a single oval canal 6 mm from the apex (verified using pre-operative CBCT scanning) were instrumented with XP-SP and F-SAF. Obtura III Max apparatus was used for root canal obturation without the use of a root canal sealer. The roots were then sectioned 6 mm from the apex and examined with a digital stereomicroscope at x25 magnification to assess the root canal fillings. The F-SAF was associated with a significantly higher (*p* < 0.01) percentage of entire adaptation of the root fillings (76%) compared to the XP-SP (57%). Furthermore, the XP-SP group was also associated with higher (*p* < 0.01) defective obturation with debris at 17% and with voids at 26%. However, the F-SAF had lower percentages of defective obturations (7% with debris and 17% with voids). The quality of obturation of oval canals instrumented using full-sequence SAF was better.

## 1. Introduction

The primary goal of root canal obturation is to achieve a hermetic seal in an instrumented and chemically cleansed root canal and its abnormalities that persist all the way to the apical terminus [1]. This is accomplished by using gutta-percha and a sealer that functions as a bacteriological prevention [2]. Gutta-percha has been successfully used as a core filler material with a sealer to occupy the instrumented and cleaned radicular space [1]. The use of thermoplasticised gutta-percha was a crucial technique for achieving significant adaptability of root canal fillings [1,2].

The literature reports a high frequency of oval root canals. The oval cross-section of the root canal dominates in the apical 6 millimetres, with mandibular incisors having the highest frequency [3]. In such canals, instrumentation of the whole radicular wall is impossible, and uninstrumented recesses may persist following the use of solid metal cored motorised endodontic instrumentation. This is because the files sculpt a round cross-sectional form in rotational motion, leaving unaffected recesses in the extremities of the oval canal’s greatest diameter [4]. It has been observed that up to 80% of radicular canal space remains unaltered [5].

Specific endodontic instruments have been developed for the treatment canals with complex architecture. Self-Adjusting File (SAF) (ReDent-NOVA Ltd., Ra’anana, Israel), TRUShape (Dentsply Sirona, Tulsa, OK, USA), Gentlefile (Gentlefile; MedicNRG, Kibbutz Afikim, Israel), XP-endo Finisher, and XP-endo Shaper are among them (FKG Dentaire, La Chaux-de-Fonds, Switzerland). 

Even if the canals are not optimally cleaned, it is preferable to thoroughly obturate them with gutta-percha and sealant for microbial control [6,7,8]. As a result, while assessing the quality of obturations, it is critical to understand the regions filled with gutta-percha or packed with debris. As a result, the goal of this study was to evaluate the adaptation of gutta-percha and defective adaptation (with voids or debris) in oval-shaped root canals instrumented utilising full-sequence SAF and XP-endo shaper plus system anatomical approaches. 

To the best of the authors’ knowledge, no literature has been published on the effect of recent instrumentation of recent recommendations issued by either manufacturer (F-SAF and XP-SP) on the quality of root canal fillings in oval canals. 

## 2. Materials and Methods

### 2.1. Sample Selection

The current study was approved by the Universitas Airlangga Faculty of Dental Medicine Health Research Ethical Clearance Commission (559/HRECC.FODM/IX/2021). In accordance with a recent study [8], to achieve a power of 0.80 at a 0.05 level of significance, a minimum of 82 (41 in each group) would be required for this study and we increased the sample size to 90 samples. A total of ninety human mandibular incisors with single and straight (curvature < 5°) [9] canals were selected from a random collection of recently extracted teeth. All samples were observed, employing a digital stereomicroscope under ×25 magnification (Labline^®^ Stock Center, Mumbai, India), to confirm absence of cracks and presence of single apical foramen. The teeth were stored in a 0.1% thymol solution at 4 °C until used for the study. For standardisation, root segments of 17 mm were obtained by sectioning crowns of the samples with a low-speed steel cutting disc (IsoMet, Buehler, Lake Bluff, IL, USA) at or below the cemento-enamel junction (CEJ). Working length (WL) was established by subtracting 1 mm from the length measured when a #10 K-file instrument was seen at the apical foramen (under stereomicroscope). The apical diameter of all the selected samples were approximately corresponding to an ISO size of 15 (confirmed by #15 K-file). Additionally, 6 mm from the apex, the long to short canal diameter ratio for all the selected root specimen was ≥2.5 (checked using buccal and lingual radiography), confirming the presence of oval canals [10].

### 2.2. Root Canal Preparation

The root canal instrumentation was performed by two endodontists experienced with the use of respective files systems (XP-SP; instrumented by A.B.) and (F-SAF; by A.M.P.). The following two file systems were used for instrumenting oval canals: rotary MaxWire XP-endo^®^ Shaper Plus sequence (FKG Dentaire, La Chaux-de-Fonds, Switzerland) and full sequence SAF system (ReDent-Nova Ltd., Ra’anana, Israel). Each file system was employed according to its manufacturer’s instructions [11,12].

#### 2.2.1. XP-endo^®^ Shaper Plus (XP-SP)

The canals in this group were instrumented with an electronically powered endomotor (XSmart plus; Dentsply/Maillefer, Ballaigues, Switzerland) and XP-endo shaper (XP-S) files were operated at 800 rpm with 1N cm torque. The canal patency of the samples was verified using a #15 k-file (Dentsply/Maillefer), and the pulp chamber was filled with 1 ml of warmed 5.25% sodium hypochlorite (NaOCl; Prime Dental Products, Mumbai, India). The XP-S tip was inserted into the canal until resistance was felt, then the file was withdrawn until it was free, and the endomotor was triggered. Long gentle strokes toward WL were used to carry the instruments. After every 5 strokes, the canal was flushed with 1 ml of preheated 5.25% NaOCl, recapitulated with #15 k-file, and filled with 1 ml of preheated 5.25% NaOCl. Following that, the canal instrumentation was resumed for the next 5 strokes or until the WL was reached. After reaching the apex, the canal was irrigated (preheated NaOCl) and the file was utilised for 15 more strokes at WL. To remove any remaining suspended material, a final flush of 4 mL of 5% NaOCl was performed. Further to the XP-S instrumentation, the XP-F (XP-finisher) file was used. The WL was determined by examining the marks on the plastic tube and adjusting the file’s stopper. The canal was filled with the irrigant (preheated NaOCl), XP-F was detached from the plastic tube, the file was placed (3–4 mm) into the canal, and the motor was turned on. The file was threaded softly into the canal. The XP-F was used for 30 s (about 30 strokes) in the canal, using moderate and gentle longitudinal motions apically to contact the whole length of the canal. The file was then removed from the canal, irrigation (preheated NaOCl) was administered, and the file was placed into the canal for another 30 s. Finally, the canal was irrigated with a final flush of 1 ml of NaOCl, 2 ml of 17% aqueous EDTA (DentWash; Prime Dental Products), and 1 ml of NaOCl. The procedure was conducted as recommended by the manufacturer. The XP-S and XP-F were used with an irrigant at a temperature of more than 37 °C to mimic body temperature. This was conducted as these files change shape when transferred from room temperature to 37 °C. 

#### 2.2.2. Full-Sequence SAF System (F-SAF)

The canal patency was verified for the canals in this group, and the WL was determined using a #10 k-file. For the coronal 3 mm of the root canal, the Pre-SAF OS was used as an orifice opener at 600 rpm and 1.5 Ncm, followed by the Pre-SAF 1 (#15/0.02; 600 rpm and 1 Ncm) and Pre-SAF 2 (#20/0.04; 600 rpm and 1.5 Ncm). The Pre-SAF 1 and 2 were employed in the canal 2–3 times in a gentle pecking motion until the WL. After each instrument, the canals were constantly irrigated with 2 mL of 5.25% NaOCl (Prime Dental Products) using a syringe and 28-G needle (RC Twents; Prime Dental Products), for a total of 6 mL. Following that, a 1.5-millimetre SAF (21 mm length) was passively inserted into the canal to the WL, and the root canal was instrumented for 4 min using a pre-programmed EndoStationTM (Redent Nova) at 5000 vibrations/min and an amplitude of 0.4 mm (ReDent Nova). According to the manufacturer’s directions, a pecking action was employed until the file reached the WL. Irrigation was carried out with 5.25% NaOCl, which was continuously supplied by the in-built VATEA peristaltic pump (ReDent Nova) at a flow rate of 4 mL/min, totalling 16 mL. At the completion of the preparation (4 min), root canal patency was verified with a #10 K-file, followed by 2 mL of 17% aqueous EDTA and a final flush of 1 mL of 5.25% NaOCl with a syringe and 28-G needle.

### 2.3. Root Canal Obturation

The Obtura III Max apparatus (Obtura Spartan, Fenton, MO, USA) was arranged as directed by the manufacturer. Obturations in both groups were accomplished with silver injection needles of 23 gauge, with a silicone stop installed 2–5 mm from the working length. During obturation, the thermoplasticised GP was injected twice independently. The needle was first entered in the apical direction until it was bonded to the canal wall, and then the thermoplasticised GP, which had been heated to 185 °C in the delivery system, was injected. The needle was removed after injecting a few millimetres of GP at the tip of the preparation. To avoid GP adhesion, the softened GP in the apical portion was vertically condensed to the apex with an alcohol-dipped hand plugger (Dentsply Maillefer, Switzerland). The remaining root canal was then backfilled in increments until the GP was discovered in the cervical portion of the root.

### 2.4. Sectioning and Analysis

The roots of both groups were sectioned horizontally to generate 1-millimetre-thick slices using a diamond coated saw with a 0.4-millimetre-thick disc (Isomet 1000; Buehler, Lake Buff, IL, USA) and continuous water cooling. The slices were cut 6 mm from the apex. The samples were not polished in order to prevent debris from forming and being stuck in non-filled regions of the root canals. Each slice was examined coronally using a stereomicroscope at ×30 magnification. The slices were photographed using a digital camera (AM423x dinoEye digital eyepiece) placed on the stereomicroscope.

### 2.5. Area-Metric Analysis

**Figure 1:**
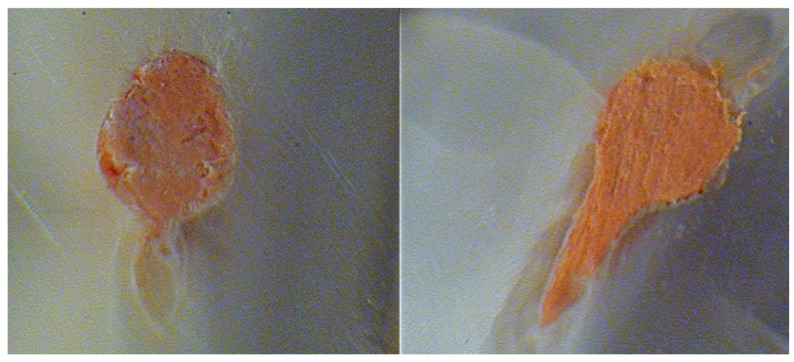
Representative images of the section of the oval root canals at 6 mm from the apex after root canal instrumentation by the tested adaptive file sequences. (**Left**)—NFM associated with debris after XP-endo shaper plus instrumentation. (**Right**)—Entire adaptation of the root filling after full-sequence SAF instrumentation.

Motic Image Plus software was used to analyse each root slice (Motic China Group, Guiyang, China). The proportion of the area of the canal with no filling material was one of the criteria examined for each slice (NFM). The entire perimeter of the canal (PC) and the area of the root-filled canal were used to determine this (RFC). RFC was subtracted from PC, and the result was divided by PC (NFM = [PC − RFC]/PC). An evaluator who was blinded to the groups measured each of the 90 (*n* = 45 each group) root slices separately. The slices were assessed for complete adaptation of root fillings and defective adaptation of root fillings. The slices were subsequently assessed for NFM with debris (white opaque material that filled the region that the obturating material failed to fill) or NFM with voids (no presence of debris but the obturating material failed to fill the area) for the defective adaptation of root fillings (Figure 1).

## 3. Results

When comparing two adaptive instrumentation methods for root canals with oval cross sections, a full-sequence SAF demonstrated complete adaptation in 76% (34/45) of the samples and defective adaptation of root canal fillings in 24% (11/45). This was significantly superior (*p* < 0.01) to the XP-endo shaper plus, which was related with 57% (26/45) of entire adaptation and 42% (19/45) of defective adaptation of root fillings, respectively (Table 1). The defective adaptation termed as No Filling Material (NFM) was significantly lower in the full-sequence SAF group (*p* < 0.01) (Table 1). 

## 4. Discussion

Root canal obturation is an essential milestone in the root canal treatment that intends to seal the root canal in three dimensions to prevent bacterial contamination/recontamination of the canal space. The current study assessed the quality of thermoplastic root fillings in oval canals following two adaptive root canal instrumentation procedures. The filling characteristics were evaluated by tabulating the percentages of complete adaptation and defective adaptation of the root fillings. The defective adaptation was then assessed based on whether it was NFM related with debris or NFM associated with voids. This evaluation was performed 6 mm from the apical foramen, where the root canal cross-section is more prominent oval, making instrumentation, irrigation, and obturation questionable [5].

The primary mechanical purpose of radicular preparation is to sustain the canal shape, with a continuous tapering funnel, while keeping the apical foramen as narrow as feasible. There are several anatomic complications that reflect physical restrictions that make proper root canal instrumentation, disinfection, and subsequent three-dimensional obturation difficult. Manual and motorised instruments use a similar theory of root canal filing and tend to leave more than half of the root canal area unaltered, especially in root canals with oval cross-sections [13,14,15]. Furthermore, rotary, or reciprocating files cut the radicular dentin, resulting in chips and tissue debris that are easily driven toward the isthmus or canal recesses rather than auguring coronally or becoming clogged within the instrument flutes that can be cleaned [13]. The debris packed in the canal recesses combines with the remaining pulp tissue and/or bacterial biofilm may create a mass that may prevent effective obturation [16]. When utilised to cleanse the canal irregularities of the packed material generated by motorised equipment, neither conventional irrigation techniques nor passive ultrasonic irrigation are shown to be successful [17]. 

Debris packing can be blamed for the poor disinfection and obturation of root canals treated with motorised files. In the present study, as in previous ones, the use of a sealer was avoided as it may be difficult to distinguish between the debris and the sealer [18,19]. The root canal sealer when used, fills the gaps between the thermoplasticised gutta-percha and radicular dentin walls. Obturation was conducted without a sealer in the current study, and inadequate obturation was assessed using no filling material (NFM) associated with either debris or voids. In samples, the existence of voids without material was predicted, implying that once the canal has been cleansed of debris, the sealant may flow into such areas. However, if the area was tightly packed with debris, sealer penetration might be compromised [18,20].

Both file systems tested in this investigation were designed to handle the 3D shape of root canal systems with any cross-section. To the best of the authors’ knowledge, no literature on the effect of the use of the XP-endo shaper plus and full-sequence SAF for obturation quality has been published. Nonetheless, studies have been published following the usage of SAF, notwithstanding the manufacturer’s new recommended sequence [18,19].

The SAF trumps rotary files in terms of adaptability to root canal fillings. This might be owing to the file’s exceptional adaptability to the root canal cross-section and continuous irrigation, both of which provide increased cleaning and shaping possibilities. When compared to other files, these are more effective in addressing the perimeter of the root canal, resulting in better cleaning of irregularly shaped root canals [21,22,23,24]. Due to debris accumulation in the canal irregularities, the root canal filling material cannot flow and establish close contact with the radicular dentine. The SAF reduces the amounts of debris packed in the canal recesses during root canal instrumentation, resulting in better obturation quality [25].

In their recent analysis, Schäfer et al. discovered that utilising K-Flexofiles, Mtwo, Reciproc, or WaveOne files had no effect on the obturation quality of thermoplasticised gutta-percha. The study’s conclusions might be correct in terms of the circular cross-sections of root canals employed for testing [26]. Oval canals are fairly frequent, despite the fact that a random series of 2D periapical radiographs would not disclose this. The mentioned motorised files are more successful when instrumenting circular root canals, but not when treating oval canals. In the case of oval canals, these files leave almost 40–60% of the root canal regions untouched by their instrumentation [27]. This is not what a dentist anticipates or intends while conducting root canal therapy in such roots. The SAF system was found to be better while treating such canals. This is not a flawless solution, but it is a lot closer to what the operator is looking for when conducting root canal therapy in such canals [13].

The XP-endo Shaper is a snake-like file that generates a space that symbolises the “envelope of motion” of the spinning file. As its motion envelope is dynamic and may contract and expand as needed in a particular canal, the XP-endo Shaper file can adapt to any cross-sectional shape of root canals [15,28]. The XP-endo Shaper differs from typical rotary files in its operation. Although NiTi rotary files have become more flexible over time, they still have a fixed core shape and taper, making them more likely to produce an area space that resembles a “circular bore.” This works effectively in narrow canals with a circular cross-section, but it has not worked well in irregularly shaped or oval root canals [15,28]. The circular whip-shape XP-endo Finisher file (#25/0.00) was first supplied as a compatible tool that could be used after any file system following root canal preparation. This file is intended to clean up the complex morphologies and difficult-to-reach regions of the root canal system [29]. 

A new manufacturer’s procedure that combines the shaper and finisher files is the XP-endo shaper plus sequence. When used alone, these two files have been claimed to be superior to standard motorised files in terms of shaping, and when combined, the result is expected to be much better.

The XP-endo shaper plus sequence is employed with intermittent irrigation, utilising a syringe and needle between file applications, which is a distinction between the two file systems that were tested in the present study. Both the file systems are moved in the canal with repeated pecking motions, thus resulting in continuous agitation of the irrigant in agitation of the irrigant. Nevertheless, in the SAF protocol, on the other hand, irrigation is continuous, since the irrigant is supplied into the root canal through the hollow file throughout the procedure. It is possible that constant irrigation, which moves material coronally and out of the canal, helps to avoid debris collection. This could be one of the possible reasons for better adaption of the root canal fillings in the F-SAF group [13,14].

In the current study, the use of a sealer was avoided for experimental reasons to allow differentiation between the debris and voids defective obturation [20]. This should not be taken as recommendation to use no sealer in the clinical situation as sealer is essential to improve the obturation of all root canal filling methods. 

The present study has some limitations. First, the study used root sections at a single level, if they represent the 3D obturation of the whole canal. Contrast-enhanced micro-CT [30] may better represent the defects in obturation of the entire root canal space, yet this was beyond the scope of the present study. The root canal instrumentation was conducted by two endodontists, each familiar with one of the file systems. Nevertheless, performing the obturation also by two individuals should be considered as a limitation, as it may have potentially introduced some bias. This should be avoided in future studies. 

## 5. Conclusions

Within the constraints of the current study, it is possible to conclude that full-sequence SAF instrumentation results in cleaner root canals with less debris in canal irregularities and better adaptation of thermoplastic gutta-percha compared to the recent recommendation of XP-endo shaper plus sequence.

## Figures and Tables

**Table 1:** Quality of obturation at 6 mm from the apex after instrumentation by the two files tested.

Group.	Entire Adaptation	NFM with Debris	NFM with Voids	Total
XP-endo shaper plus	26/45 (57%)	7/45 (17%)	12/45 (26%)	45/45 (100%)
Full-sequence SAF	34/45 (76%)*	3/45 (7%)*	8/45 (17%)*	45/45 (100%)

The values marked with (*) exhibited significantly better results exhibiting a *p* < 0.01.

## Data Availability

There are no data other than the that reported in the study available.
